# Genomic prediction for rust resistance in pea

**DOI:** 10.3389/fpls.2024.1429802

**Published:** 2024-07-23

**Authors:** Salvador Osuna-Caballero, Diego Rubiales, Paolo Annicchiarico, Nelson Nazzicari, Nicolas Rispail

**Affiliations:** ^1^ Institute for Sustainable Agriculture, Spanish National Research Council (CSIC), Cordoba, Spain; ^2^ Research Centre for Animal Production and Aquaculture, Spanish National Research Council (CREA), Lodi, Italy

**Keywords:** DArTseq, Genotype x Environment Interaction, genomic selection, *Pisum* spp., *Uromyces pisi*

## Abstract

Genomic selection (GS) has become an indispensable tool in modern plant breeding, particularly for complex traits. This study aimed to assess the efficacy of GS in predicting rust (*Uromyces pisi*) resistance in pea (*Pisum sativum*), using a panel of 320 pea accessions and a set of 26,045 Silico-Diversity Arrays Technology (Silico-DArT) markers. We compared the prediction abilities of different GS models and explored the impact of incorporating marker × environment (M×E) interaction as a covariate in the GBLUP (genomic best linear unbiased prediction) model. The analysis included phenotyping data from both field and controlled conditions. We assessed the predictive accuracies of different cross-validation strategies and compared the efficiency of using single traits versus a multi-trait index, based on factor analysis and ideotype-design (FAI-BLUP), which combines traits from controlled conditions. The GBLUP model, particularly when modified to include M×E interactions, consistently outperformed other models, demonstrating its suitability for traits affected by complex genotype-environment interactions (GEI). The best predictive ability (0.635) was achieved using the FAI-BLUP approach within the Bayesian Lasso (BL) model. The inclusion of M×E interactions significantly enhanced prediction accuracy across diverse environments in GBLUP models, although it did not markedly improve predictions for non-phenotyped lines. These findings underscore the variability of predictive abilities due to GEI and the effectiveness of multi-trait approaches in addressing complex traits. Overall, our study illustrates the potential of GS, especially when employing a multi-trait index like FAI-BLUP and accounting for M×E interactions, in pea breeding programs focused on rust resistance.

## 1 Introduction

Pea, *Pisum sativum* L. (2n = 2x = 14), is an important cool-season grain legume crop cultivated predominantly in temperate climates. With an annual global production exceeding 14 million tons of dry peas and 21 million tons of green peas (FAOSTAT, 2022), it holds substantial nutritional value, being a rich source of proteins, starch, fibers, vitamins, and minerals. Its symbiotic relationship with nitrogen-fixing bacteria underscores its role in enhancing soil fertility, making it a key component in sustainable cropping systems (Guo et al., 2021). Cultivars of pea are primarily inbred lines, largely homozygous, developed through several generations of self-fertilization following initial hybridization (van de Wouw et al., 2010). This breeding process is time-intensive, requiring years to yield genetically and phenotypically stable lines suitable for field trials and eventual commercialization. Hence, there is a high need for more efficient breeding strategies based on high throughput genotyping or phenotyping, to expedite the identification and development of elite lines (Annicchiarico et al., 2023).

One of the main objectives in the development of elite pea lines is the introduction of new resistance sources to pests and diseases, which are major constraints to global pea production (Rubiales et al., 2023). Therefore, disease resistance is a key focus in pea breeding programs, and significant advances have been made using marker-assisted selection (MAS) for diseases controlled by single genes. For instance, polymerase chain reaction (PCR) markers facilitate the identification of breeding lines carrying DNA polymorphisms linked to resistance against viruses such as Pea Seedborne Mosaic Virus (Grimm and Porter, 2021) and Pea Enation Mosaic Virus (Jain et al., 2013), as well as a fungal diseases like powdery mildew whose resistance is controlled by *er1*, *er2* and *Er3* genes (Fondevilla and Rubiales, 2012). However, challenges persist in managing diseases with polygenic resistance nature, including root rot (Leprévost et al., 2023), fusarium wilt (Sampaio et al., 2020), root parasitic weeds like broomrapes (Fondevilla et al., 2010), and aerial diseases such as ascochyta blight (Barilli et al., 2016) or rust (Osuna-Caballero et al., 2022).

Rust, caused by *Uromyces* spp., can reduce pea yield by up to 50%, varying with environmental conditions and the specific pathogen involved. *U. viciae-fabae* predominantly affects pea in tropical and subtropical climates, while *U. pisi* is prevalent in temperate regions, both causing significant epidemic cycles during the crop season (Singh et al., 2023). Complete resistance, defined as the plant’s ability to fully prevent infection by the rust causal agent, has yet to be identified in pea although some advances have been made in other legumes (Osuna-Caballero et al., 2024a). Measuring partial resistance in pea remains challenging due to the influence of environmental factors such as rainfall, temperature, and inoculum levels on disease prevalence in the field (Das et al., 2019). Partial resistance to *U. pisi*-induced rust is multigenic, with some quantitative trait loci (QTL) identified in biparental populations using wild relatives as resistance donors (Barilli et al., 2010; Barilli et al., 2018). In addition, genome-wide association studies (GWAS) have identified 95 DArT-seq polymorphic markers linked to rust resistance, pointing to 62 candidate genes putatively involved in resistance to *U. pisi* (Osuna-Caballero et al., 2024b). Genomic selection (GS), utilizing a wide array of genetic markers across the genome, offers a promising approach to select elite breeding lines for a complex, multigenic trait such as rust resistance.

GS, originally pioneered in livestock breeding, has expanded its utility across a diverse range of plant species, encompassing fruit and timber trees (Resende et al., 2012; Wang et al., 2023), as well as major crops such as maize and wheat (Crossa et al., 2017). GS accelerates the breeding cycle in annual inbred crops, enabling earlier selection of breeding parents based on Genomic Estimated Breeding Values (GEBV) in successive progeny generations (Lin et al., 2017). Therefore, GS approach is considered a pivotal methodology in the advancement of new crop varieties that hold significant value for farmers. The focus of GS studies has often been on GEI, to improve the GS prediction by integrating GEI effects as covariates (Tolhurst et al., 2022). This approach may include the prediction of GEBVs in different environments for lines lacking phenotypic data or only partially phenotyped. The application of GS in plant breeding varies significantly among crops and traits, depending on the trait genetic architecture and specific breeding and cultivation systems (Akdemir and Isidro-Sánchez, 2019). In pea breeding, GS has been employed to assess important agronomic traits such as thousand-seed weight, seed number per plant, and flowering date (Tayeh et al., 2015). Remarkably, prediction accuracies for traits like thousand-seed weight were as high as 0.83, underscoring the potential of GS in pea breeding, particularly when traits are relatively easy to measure and highly heritable. In addition, the size and composition of the training population, carefully selected, significantly affect prediction accuracy (Akdemir and Isidro-Sánchez, 2019). In recent research, GS has also been applied to predict pea grain yield, protein content, and morphological traits using Genotyping-by-Sequencing (GBS) data (Annicchiarico et al., 2019, Annicchiarico et al., 2020; Crosta et al., 2022, Crosta et al., 2023). These approaches yielded fairly accurate intrapopulation and interpopulation predictions for grain yield, proving to be cost-effective when considering that phenotyping costs are notably higher than genotyping costs (Annicchiarico et al., 2019). These findings support the use of GS in pea breeding programs. However, the efficacy of GS for disease resistance traits in pea is largely unknown, with information available so far only for ascochyta blight resistance (Carpenter et al., 2018). This information is particularly valuable because it could enhance the economic efficiency of GS by enabling the simultaneous selection for various key traits without additional costs - since the genotyping costs remain the same regardless of the number of selected traits, unlike the phenotyping costs.

GS requires comprehensive genotypic data, typically acquired through methods like high-density arrays, genotyping by sequencing (GBS) or reduced representation genome sequencing (RRGS). Among genome complexity reduction technology approaches, DArTSeq genotyping has emerged as a suitable genomic method for GS, genetic mapping, and population genetic studies in many plant species (Alam et al., 2018; Alemu et al., 2022). This genotyping method, which enables the generation of both SNP and SilicoDArT markers, is particularly effective for crops with large genomes abundant in repetitive sequences, such as pea (Barilli et al., 2018). It sequences regions adjacent to restriction enzyme sites, and preferentially targets coding regions over repetitive DNA by employing methylation-sensitive enzymes (Akbari et al., 2006). In GS analyses, prediction accuracy is often gauged by the correlation between predicted and observed trait values. However, beyond estimating the breeding value (BV) for entire populations, plant breeders are particularly interested in accurately predicting top-performing individuals for selection as elite cultivars or parental lines for subsequent breeding cycles (Bassi et al., 2016). GS presents a dual advantage in pea genetic improvement: it facilitates the prediction of GEBV for individuals lacking phenotypic data and enhances the precision of BV estimates for phenotyped individuals, especially for traits that are challenging to measure (Rubiales et al., 2021). This is achieved by integrating trait data from multiple environments and/or years with genotypic information. Moreover, leveraging molecular data for BV estimation offers inherent advantages over traditional pedigree-based approaches (Hayes et al., 2009). Molecular data provide a ‘realized relationship matrix,’ reflecting the actual genetic relationships among individuals, as opposed to the ‘expected values’ used in pedigree-based matrices, where relative individuals are assumed to have average and equal genetic contributions (Crossa et al., 2010). This nuanced understanding of genetic relationships afforded by molecular data underpins the enhanced accuracy of GS, making it a transformative tool in modern plant breeding.

Assessments of different models usable for genomic selection have not revealed a single model that always outperform the others, since model performance depends on the number of genomic regions influencing a trait and the magnitude of its effects (Habier et al., 2011; Heffner et al., 2011). Furthermore, different models make assumptions that may or may not match the genetic architecture of the trait. The main objective of this study was to provide a comprehensive evaluation of GS models for rust resistance in peas, tailoring our approach to align with the practical demands of current pea breeding programs (Annicchiarico et al., 2023). Utilizing genotypic data from Rispail et al. (2023) and phenotypic data for resistance to *U. pisi* from Osuna-Caballero et al. (2022), we trained and validated several GS models. Our comparison verified the influence of multi-trait indices on their predictive accuracy and investigated the role of GEI in the context of field condition predictions, using three cross-validation schemes of high relevance and applicability for GS equations in plant breeding. This methodical approach allowed us not only to assess the efficacy of different GS models in a breeding context but also to explore how the integration of complex data, such as multi-trait indices and environmental interactions, can optimize predictive accuracies, thereby crucially supporting the deployment of GS strategies for enhancing rust resistance in pea.

## 2 Material and methods

### 2.1 Plant material

The pea panel utilized in this study comprise an extensive collection of 324 accessions of *Pisum* spp., encompassing a diverse range of genetic material, including wild relatives, landraces, cultivars, breeding lines, and unidentified genotypes sourced from various continents. The selection of these genotypes was carried out meticulously, with an aim to cover a broad spectrum of genotypic and phenotypic variance, as highlighted by Rispail et al. (2023). This approach ensures a comprehensive representation of the *Pisum* genera, capturing the genetic diversity across the three main species – *P. sativum*, *P. fulvum*, and *P. abyssinicum* – as well as the *P. sativum* subspecies, namely *sativum*, *arvense*, *jomardi*, *elatius*, and *humile*. The selection was also based on specific criteria including historical resistance performance, genetic diversity, and unique phenotypic profile that are potentially linked to disease resistance and favorable agronomic traits (Rispail et al., 2023). The inclusion of genotypes collected worldwide not only adds to the genetic mixture but also allows for the examination of GEI effects.

### 2.2 Phenotyping and statistical analysis

The pea panel was evaluated under rain-fed conditions in three autumn-sown environments in Cordoba, southern Spain. These environments are referred to as Cordoba 2018 (DS-2018), Cordoba 2019 (DS-2019), and Cordoba 2020 (DS-2020). According to the Köppen–Geiger classification system, this location represents the hot dry-summer Mediterranean climate, a common form of the Mediterranean climate characterized by hot, dry summers and mild, wet winters (Kottek et al., 2006).

For each growing season, the experiment was conducted in an alpha lattice design with 19 blocks replicated 3 times. Each incomplete block contained 17 experimental units (i.e., accessions) plus two control plots using cv. Cartouche as susceptible rust check. The trial procedures and evaluations are detailed in Osuna-Caballero et al. (2022) and a graphical representation of the experimental design to facilitate its visualization is depicted in **Supplementary Figure 1**. The recorded trait was disease severity (DS), expressed as the proportion of rust pustules covering the whole plants in the experimental unit. For each environment a one-way mixed effect model was applied to analyze the variance components following the formula:


$$y_{ijk}=\;\mu +\;\alpha_{i}+\;\gamma_{j}+{\left(\gamma \tau \right)}_{jk}+\epsilon_{ijk}$$

where $y_{ijk}$ is the DS value of the *i* ^th^ genotype (*i* = 1, 2, …, 324) in the *k* ^th^ incomplete block (*k* = 1, 2,., 19) of the *j* ^th^ replicate (*j* = 1, 2, 3); $\alpha_{i}$ is the random effect of the *i* ^th^ genotype; $\gamma_{j}$ is the fixed effect of the *j* ^th^ complete replicate; ${\left(\gamma \tau \right)}_{jk}$ is the random effect of the *k* ^th^ incomplete block nested within the *j* replicate; and $\epsilon_{ijk}$ is the error associated to $y_{ijk}$ . Broad-sense heritability (H^2^) was estimated using the formula from Toker (2004), defined as the ratio of genotypic variance to phenotypic variance. This approach also facilitated the calculation of the predicted means for each genotype, named Best Linear Unbiased Predictors (BLUPs), following the methodology of DeLacy et al. (1993) which were used in subsequent analyses.

In addition, a second linear mixed model was applied using a multi-environment trial (MET) approach. The two-way model with interaction effect used to analyze MET data followed the formula:


$$y_{ilk}=\;\mu +\;\alpha_{i}+\;\tau_{l}+{\left(\alpha \tau \right)}_{lk}+\gamma_{lk}+\epsilon_{ilk}$$

In this case, $\tau_{l}$ is the fixed effect of the l^th^ environment (*l* = DS-2018, DS-2019, DS-2020); ${\left(\alpha \tau \right)}_{lk}$ is the interaction random effect of the i^th^ genotype with the l^th^ environment; $\gamma_{lk}$ is the fixed effect of the k^th^ block within the l^th^ environment. This model enabled the estimation of BLUPs for DS across the three environments, representing the GEI-independent component (DS-joint). Heritability in the MET model was calculated as the genotypic variance divided by the sum of the genotypic variance, GEI variance, and residual variance.

The entire pea panel was also inoculated with *U. pisi* to assess rust symptoms in controlled conditions (CC). This experiment allowed the evaluation of four traits related to rust disease in pea seedlings: (i) final infection frequency (IF), measured as the number of pustules per cm² of leaf, counted at 14 day post-inoculation; (ii) the area under the disease progress curve (AUDPC) based on the daily IF scores from day 7 to day 14 post-inoculation; (iii) infection type (IT), classified according to Stakman et al. (1962) and (iv) DS, quantified in controlled conditions as the percentage of tissue damaged by pustules.

Each accession underwent two evaluation rounds through inoculation, with three inoculations performed using a randomized complete block design (RCBD) leading a total of six replicates per accession. Data quality control was performed individually for each trait through graphical inspection of residuals to assess normality, homogeneity of variance, and to detect outliers as described in Osuna-Caballero et al. (2022). To ensure residuals normalization and variance stabilization, arcsine transformation was applied on DS while square root transformation was applied for AUDPC and IF values. The model applied to assess the variance components in CC was similar to the previous one-way mixed effect model but without considering the block effect ${\left(\gamma \tau \right)}_{jk}$ in the formula. Therefore, four models were fitted, one per evaluated trait in CC. This allowed the calculation of the genetic, phenotypic and error variance, allowing the estimation of H^2^ and BLUPs. The BLUPs estimated for each trait collected under CC served as phenotypic data for computing a multi-trait index (FAI-BLUP) based on factor analysis and ideotype-design proposed by Rocha et al. (2018), and for subsequent genomic prediction assessments. The genetic correlation (r_g_) for genotype rust responses across traits and environments was estimated according to Howe et al. (2000).

Finally, GEI variation was dissected in more details using an additive main effects and multiplicative interaction (AMMI) analysis (Gauch et al., 2008). This approach is particularly valuable for unravelling patterns of GEI because the AMMI model decompose them into principal component axes, facilitating a clear interpretation of the interaction structure (Olivoto et al., 2019). Therefore, it was applied to determine the range of genotype stability and adaptability among the nine most resistant and the three most susceptible lines identified in the study by Osuna-Caballero et al. (2022). All phenotypic models were fitted using the ‘metan’ package (Olivoto and Lucio, 2020) in R software version 4.2.2 (R Core Team, 2021).

### 2.3 Genotyping and data filtering

The pea core collection was genotyped using the DArTSeq approach by DiversityArray Ltd, Australia. For this process, the third compound leaves from twenty two-week-old seedlings of each accession, grown under controlled conditions, were harvested. These samples were pooled, flash-frozen in liquid nitrogen, and subsequently lyophilized. DNA extraction was then carried out following the method prescribed by Diversity Arrays P/L, Canberra, Australia (https://www.diversityarrays.com). The extracted DNA was adjusted to a concentration of 20 ng/µl prior to DArT marker analysis. This analysis was conducted using the high-density Pea DArTseq 1.0 array, which consists of 50,000 markers and is specially adapted for wild *Pisum* spp. accessions. The genotyping process involved complexity reduction using PstI-MseI restriction enzymes, followed by library construction, amplification, and Illumina sequencing. These steps were performed by Diversity Arrays Technology Pty Ltd, Canberra, Australia, as detailed in Barilli et al. (2018). The DArTSeq sequence analysis yielded two sets of markers: Single Nucleotide Polymorphisms (SNPs) and presence-absence sequence variants (Silico-DArT). Marker density has a positive impact over predictive abilities in GS (Jannink et al., 2010). Therefore, Silico-DArT markers was the genetic information used in the GS analysis as they yielded a higher number of polymorphic variants (66,643 Silico-DArT vs 55,269 SNP markers; Rispail et al., 2023).

**Figure 1:**
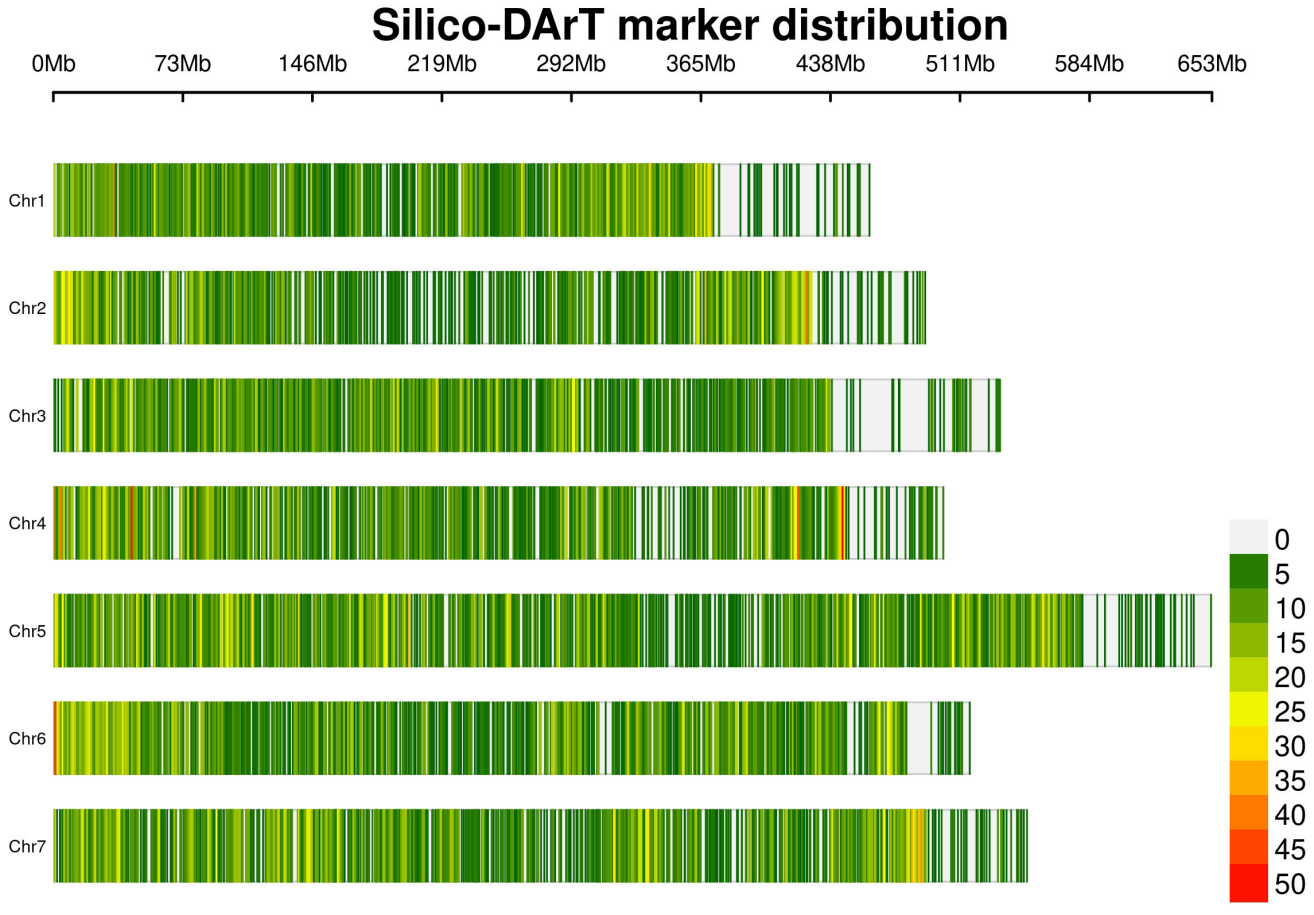
Physical distribution and density of the 26,045 Silico-DArT markers across the seven chromosomes of the pea genome.

Therefore, data cleaning was performed on the Silico-DArT dataset, obtaining a total of 26,045 markers ready to use for GS with an excellent genome coverage (**Figure 1**). This process was undertaken to eliminate low-quality and non-polymorphic markers, as described by Rispail et al. (2023). Markers exhibiting more than 20% missing data, a minor allele frequency (MAF) below 5%, and heterozygosity exceeding 0.1% were excluded from the analysis. Missing data were imputed using the Singular Value Decomposition (SVD) method, adhering to the recommendations of Nazzicari et al. (2016).

### 2.4 Genomic regression models and data configurations

Genome-enabled predictions in this study were based on 26,045 Silico-DArT markers. We focused on three genomic prediction models known for their predictive ability in legume species, particularly in relation to pea traits: Ridge regression BLUP (rrBLUP), Bayesian Lasso (BL), and Genomic BLUP (GBLUP), as identified in previous comparisons (Annicchiarico et al., 2017a; Carpenter et al., 2018).

The rrBLUP model, proposed by Meuwissen et al. (2001), assumes a common variance across all loci, making it suitable for traits influenced by many minor genes. Its linear mixed additive model equation is:


y=μ1+Gu+ϵ

where ***y*** is the vector of observed phenotypes, *μ* is the mean of ***y***, **1** refers to the vector of ones allowing for the inclusion of the *μ*, ***G*** is the genotype matrix (i.e., {0, 1} for absence/presence sequence variants Silico-DArT markers), $u∼N(0,I\sigma^{2}u)$ is the vector of marker effects, and $\epsilon ∼N(0,I\sigma^{2}\epsilon )$ is the vector of residuals. The solution for the marker effects **u**, utilizing standard ridge-regression methods, is obtained through the equation:


$$u={\left(G'G+\lambda I\right)}^{(-1)G'y}\;$$

where $\lambda =\sigma_{e}^{2}/\sigma_{u}^{2}$ is the ridge parameter, representing the ratio between the residual variance and the variance of marker effects (Searle et al., 2006) estimated by a REML method implemented by a spectral decomposition algorithm (Hyun et al., 2008). Given the vector of marker effects, it is then possible to predict phenotypes and GEBV.

The GBLUP model follows a similar equation to rrBLUP but uses a marker-based genomic relationship matrix (GRM) instead of the marker matrix (Hayes et al., 2009). The kinship coefficient matrix based on Silico-DArT markers were computed following the Astle and Balding (2009) methodology using the “statgenGWAS” R package (van Rossum and Kruijer, 2023). This model was also trained after incorporating the marker x environmental (M×E) effect matrix as covariate to evaluate its influence over the predictions. The R-script followed to fit single-environment, across-environment, and M×E GBLUP models were described in Lopez-Cruz et al. (2015) using the “BGLR” R package (Pérez and de los Campos, 2014).

Bayesian Lasso (BL) model permits different effects and variances for markers, typically few with large effects (Wang et al., 2018). This model assigns prior densities to marker effects and induce various types of shrinkage (Park and Casella, 2008). The BL system was solved via Gibbs sampling approach (Casella and George, 1992) with proper iteration count (12,000 repetitions in our configuration) and burn-in period (1,200 repetitions in our configuration) to ensure convergence. In addition, the kinship matrix was added to the model as a fixed (i.e., flat prior) component to account for population structure.

Predictive ability (r_ab_) of these genome-enabled models for rust traits in the pea panel was assessed using the R package “GROAN” (Nazzicari and Biscarini, 2022). r_ab_ was estimated as Pearson’s correlation between observed and predicted phenotypes, following three cross-validation (CV) strategies:

1. Single trait and intra-environment cross-validation [CV0], training and testing each trait per environment. In this intra-environment prediction scenario, the predictive ability of GS models was assessed by a standard within-location 10-fold cross-validation. Each analysis was performed 50 times, reporting the average result, to ensure numerical stability.
2. Single trait and cross-environment validation [CV1], predicting known lines (including in the training) over a different environment used in the training, by splitting the training data in a 90/10 fashion as done for intra-environment predictions. The whole procedure was repeated 50 times for numerical stability.
3. The third CV configuration [CV2] also consists in single trait and across-environment cross-validation but predicting new lines (not included in the training) in an untrained environment.

Overall, we assessed 11-model configurations represented by combinations of three genomic prediction models (rrBLUP, GBLUP or BL) in which M×E interaction is evaluated in GBLUP model and three CV procedures with one marker data set (Silico-DArT).

Finally, the accuracy (r_ac_) of these models was estimated from r_ab_ and the square root of broad-sense heritability on an entry mean basis in the validation environment (H), following Lorenz et al. (2011): $r_{ac}=\frac{r_{ab}}{H}$

## 3 Results

### 3.1 Phenotypic data

**Figure 2:**
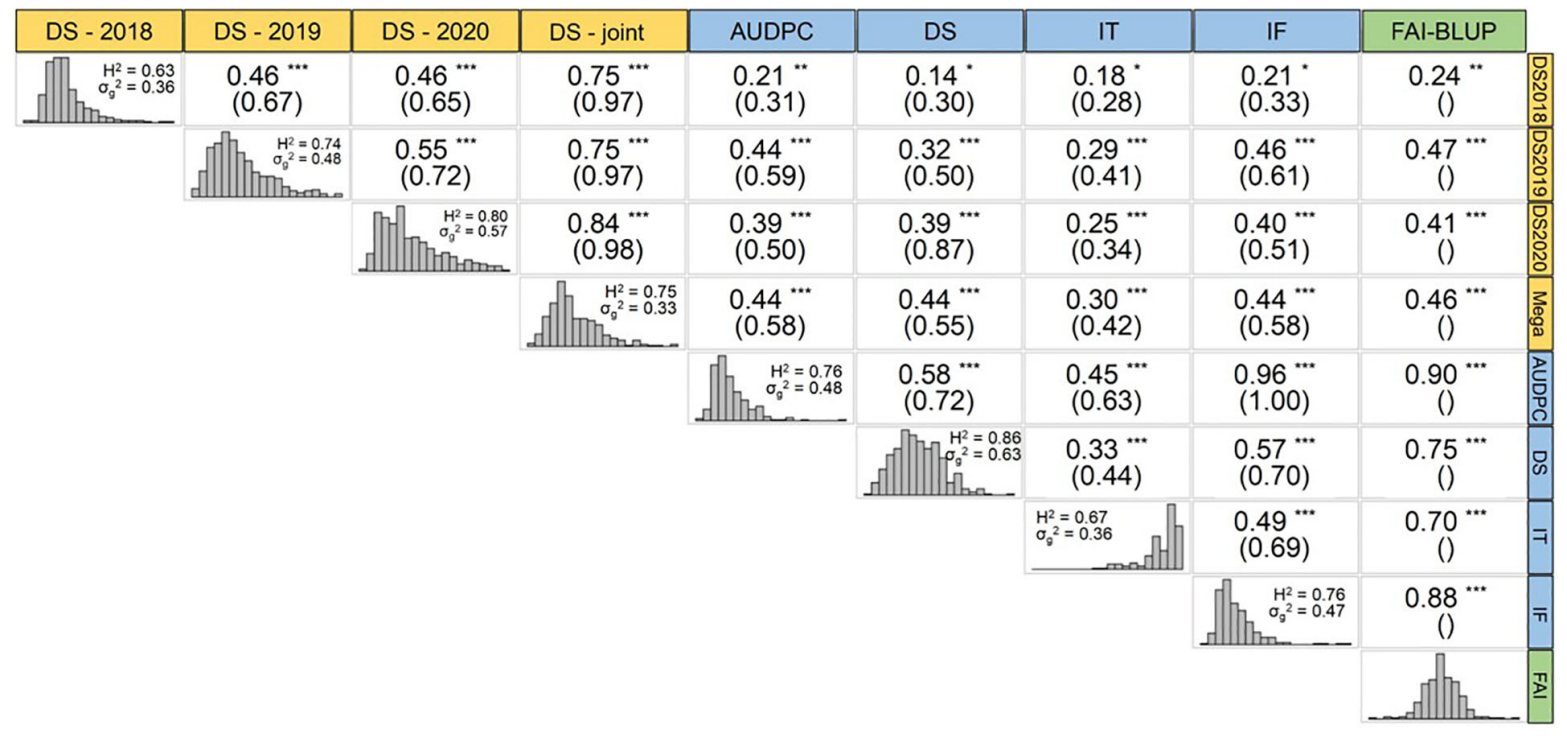
Trait correlation between field (yellow), controlled conditions (blue) and FAI-BLUP index (green). Phenotypic correlation (r_p_) and genotypic correlation (r_g_) under brackets are depicted in each trait’s intersection. Each trait shows its distribution, heritability, and genetic variance. *, ** and *** represent the significance of the r_p_ at 0.05, 0.01 and 0.001, respectively.

The phenotypic data revealed consistent patterns across different years under field condition and between traits measured under controlled conditions as shown in Osuna-Caballero et al. (2022). Here, we further explored the phenotypic correlation (r_p_) between the FAI-BLUP index, and the single traits measured under controlled and field conditions, as well as their genetic correlations (**Figure 2**). Notably, the integration of traits measured under controlled conditions into the FAI-BLUP index improved Pearson correlations between traits. Consequently, the r_p_ values between FAI-BLUP and DS in 2018 (DS-2018), 2019 (DS-2019), 2020 (DS-2020), and DS-joint, were 0.24, 0.47, 0.41, and 0.46, respectively. These values were higher than those between DS in controlled conditions and DS-2018 (r_p_ = 0.14), DS-2019 (r_p_ = 0.32), DS-2020 (r_p_ = 0.39), and DS-joint (r_p_ = 0.44). In addition, the correlations between FAI-BLUP and field data were also higher than those estimated between any other single trait measured under controlled conditions (AUDPC, IT and IF) and the corresponding field data (**Figure 2**).

Under controlled conditions, we also observed that the FAI-BLUP index correlated more strongly with individual traits and was the only parameter with a non-skewed distribution (**Figure 2**). The correlation between AUDPC and FAI-BLUP was the highest (r_p_ = 0.90), while the correlation between IT and FAI-BLUP was comparatively lower but still robust (r_p_ = 0.70).

The genetic correlation, r_g_, which assesses the extent to which the same genes influence a trait across different environments (such as different field seasons or traits under controlled conditions), also revealed differences between traits/environments. To calculate the r_g_, the formula needs the variance components (i.e., reps or blocks). Therefore, it was not possible to compute the r_g_ between FAI-BLUP and the other parameters since FAI-BLUP represents a single value for each genotype, obtained from the merged CC parameters. Nonetheless, the highest genetic correlation was observed between DS-2020 and DS-2019 (r_g_ = 0.72), suggesting that rust severity in those years was influenced by similar genetic factors. By contrast, genetic correlations were generally lower between traits measured under controlled conditions and in the field, with the notable exception of DS, which exhibited a high genetic correlation (r_g_ = 0.87) with DS-2020 (**Figure 2**).

**Figure 3:**
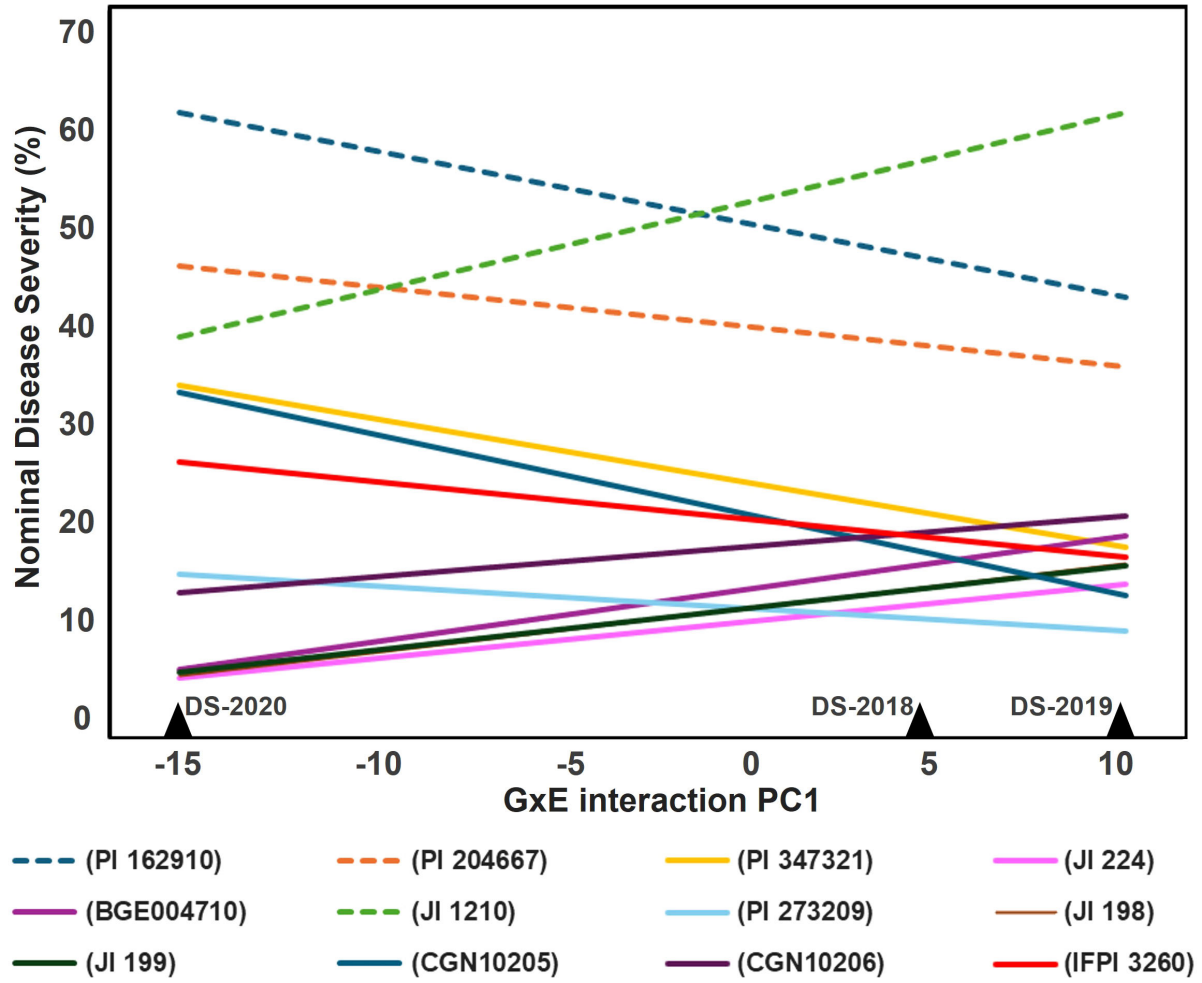
Estimated nominal disease severity (DS%) of nine most resistant (straight lines) and three susceptible (dashed lines) accessions based on FAI-BLUP index along the GEI PC1 axis.

The AMMI analysis of DS percentages revealed that the first GEI principal component axis (PC1) was highly significant (P< 0.001), explaining 80% of the GEI variance (**Supplementary Table 1**). This axis notably distinguished the 2020 field season from the two preceding years in terms of genotype responses, as depicted in **Figure 3**. The AMMI model illustrated pronounced GEI interactions of cross-over type (i.e., characterized by a range change between the resistant and susceptible lines across the different environments). In the 2019 field environment there was some increase in line variation (**Figure 3**). Importantly, resistant, and susceptible accessions formed two clusters that did not intersect across environments, indicating consistency in their response patterns, (**Figure 3**). Specifically, the lines JI 224, PI 273209, JI 199, and CGN 10206 exhibited a distinct advantage in terms of resistance and stability across these relatively contrasting environments (**Figure 3**).

### 3.2 Genome-enabled modelling

#### 3.2.1 Predictive abilities of rrBLUP, BL and GBLUP under CV0 scenario

We assessed the intra-environmental ability of three genomic prediction models (rrBLUP, BL and GBLUP) trained with a Silico-DArT marker dataset to predict rust disease in pea across controlled and field conditions. Based on average predictive ability (r_ab_) values obtained for each trait (**Table 1**), rrBLUP and BL showed similar predictive abilities for all traits. Under controlled conditions, predictive abilities (r_ab_) of BL varied from 0.569 for IF to 0.635 for FAI-BLUP. In this scenario, r_ab_ of the GBLUP model were slightly lower as shown in **Table 1**. The highest predictive ability of the three models was reached for the FAI-BLUP trait.

**Table 1:** Intra-environment predictive ability of Ridge regression BLUP (rrBLUP), Bayesian Lasso (BL) or Kernel Genomic BLUP (GBLUP) models trained with a Silico-DArT dataset to predict pea rust disease parameter in controlled or field environments.

	Controlled Conditions					Field Conditions			
Model	AUDPC	IF	IT	DS-CC	FAI-BLUP	DS-2018	DS-2019	DS-2020	DS-joint
**rrBLUP**	0.602	0.572	0.579	0.601	0.633	0.258	0.544	0.308	0.460
**BL**	0.602	0.569	0.571	0.604	0.635	0.261	0.541	0.302	0.459
**GBLUP**	0.576	0.510	0.544	0.590	0.633	0.270	0.558	0.310	0.446

For the field data, the predictive abilities of the three models were notably lower. In this case, the GBLUP model achieved the highest predictive ability for each single environment. Its ability to predict DS-joint (r_ab_ = 0.446), which includes the overall environmental variance, was nonetheless slightly lower than rrBLUP and BL (**Table 1**).

#### 3.2.2 Predictive abilities of rrBLUP, BL and GBLUP under CV1 scenario

The genomic selection models were also trained and tested across different environments, to evaluate their ability and accuracy in predicting rust disease in pea. Under the CV1 scenario, moderate predictive abilities were obtained for all three models when they were trained with DS values obtained under controlled conditions and tested on the DS-joint data as shown in (**Table 2**. In this case, the highest predictive ability was reached by GBLUP (r_ab_ = 0.382). Interestingly, training models with the FAI-BLUP index dataset and testing them on DS-joint data improved the model predictive abilities with GBLUP exhibiting the highest predictive ability (r_ab_ = 0.500) and accuracy (r_ac_ = 0.577).

**Table 2:** Cross-environment [CV1] predictive ability (r_ab_) and predictive accuracy (r_ac_) of pea rust across different traits and two environments estimated with Ridge regression BLUP (rrBLUP), Bayesian Lasso (BL) or Kernel Genomic BLUP (GBLUP) model trained on a Silico-DArT marker dataet.

Training set	Test set	*r*_ab_			*r*_ac_		
		rrBLUP	BL	GBLUP	rrBLUP	BL	GBLUP
DS-CC	DS-joint	0.378	0.369	0.382	0.436	0.426	0.441
FAI-BLUP	DS-joint	0.419	0.402	0.500	0.484	0.464	0.577
DS-2018	DS-2019	0.498	0.491	0.605	0.579	0.571	0.703
DS-2018	DS-2020	0.384	0.411	0.465	0.430	0.460	0.520
DS-2019	DS-2018	0.357	0.358	0.369	0.450	0.451	0.465
DS-2019	DS-2020	0.463	0.465	0.503	0.518	0.520	0.562
DS-2020	DS-2018	0.357	0.386	0.388	0.450	0.486	0.489
DS-2020	DS-2019	0.605	0.596	0.627	0.703	0.693	0.730

For the field data under CV1 scenario, models trained on DS-2018 and tested on DS-2019 exhibited high predictive abilities and accuracies, especially for GBLUP (**Table 2**). Conversely, models trained on DS-2019 and tested on DS-2018, led to the lowest predictive abilities and accuracies. In fact, GS models tested across-environment yielded lower predictive abilities when DS-2018 was used to test the trained data. By contrast, the highest predictive abilities and accuracies were observed after training the models on DS-2020 and testing them on DS-2019 (**Table 2**). Once again, GBLUP outperformed rrBLUP and BL, exhibiting predictive ability and accuracy of 0.627 and 0.730 respectively (**Table 2**).

#### 3.2.3 Predictive abilities of rrBLUP, BL and GBLUP under CV2 scenario

In the last cross-validation scheme (CV2), the GS models were trained on one set of data and validated on a different, untrained environment, simulating the prediction of new pea lines performance. When the models were trained on controlled condition DS and validated on DS-joint, the predictive abilities were moderate, with GBLUP exhibiting a slightly higher ability (r_ab_ = 0.332) compared to rrBLUP and BL (**Table 3**). Training on FAI-BLUP and validating on DS-joint data improved the model predictive abilities with GBLUP displaying again the highest ability and accuracy (**Table 3**).

**Table 3:** Cross-environment [CV2] predictive ability (r_ab_) and predictive accuracy (r_ac_) of pea rust traits estimated with Ridge regression BLUP (rrBLUP), Bayesian Lasso (BL) or Kernel Genomic BLUP (GBLUP) model trained on a Silico-DarT marker dataset.

Training set	Validation set	*r*_ab_			*r*_ac_		
		rrBLUP	BL	GBLUP	rrBLUP	BL	GBLUP
DS-CC	DS-joint	0.329	0.319	0.332	0.380	0.368	0.383
FAI-BLUP	DS-joint	0.331	0.400	0.427	0.382	0.462	0.493
DS-2018	DS -2019	0.377	0.330	0.519	0.438	0.384	0.603
DS -2018	DS -2020	0.223	0.217	0.295	0.249	0.243	0.330
DS -2019	DS -2018	0.198	0.197	0.177	0.250	0.220	0.223
DS -2019	DS -2020	0.326	0.307	0.352	0.365	0.384	0.394
DS -2020	DS -2018	0.198	0.204	0.201	0.250	0.236	0.253
DS -2020	DS -2019	0.479	0.435	0.510	0.557	0.502	0.593

For the field datasets, models trained on DS-2018 and validated on DS-2019 showed notable predictive abilities and accuracies, particularly GBLUP, while validating these models with DS-2020 decrease predictive abilities and accuracies. Training models with DS-2019 led to low predictive abilities and accuracies. In addition, to validate the models with DS-2018 data set produced the lowest predictive abilities and accuracies in this case. Conversely, training models on DS-2020 and validating them with DS-2019 exhibited again the highest predictive ability (r_ab_ = 0.510 for GBLUP) and accuracy (r_ac_ = 0.593 for GBLUP) within this validation scenario (**Table 3**).

#### 3.2.4 Influence of the marker x environment interaction in the GBLUP prediction

To improve the predictive abilities and accuracies of the GBLUP model, we incorporated the M×E interaction matrix as covariate within two cross-validation strategies: CV1 and CV2 (**Table 4**). When models were trained on controlled condition DS and tested on DS-joint dataset, the inclusion of the M×E interaction improved the predictive abilities and accuracies under both CV1 and CV2 scenarios, reaching r_ab_ values of 0.457 and 0.443 and r_ac_ values of 0.528 and 0.511, respectively. The addition of the M×E covariate also improved the predictive abilities and accuracies of GBLUP models trained on FAI-BLUP index and validated on DS-joint (reaching r_ab_ value of 0.500 and 0.465 and r_ac_ values of 0.578 and 0.537 for CV1 and CV2 scenarios, respectively (**Table 4**). Overall, FAI-BLUP was found to be the best training-controlled condition trait to predict accession response to rust in the field.

**Table 4:** Predictive ability (r_ab_) and predictive accuracy (r_ac_) fitting the Kernel Genomic BLUP (GBLUP) model with the effect of the marker x environment interaction (M×E) as covariate in two Cross-Validation strategies.

Training set	Validation set	*r*_ab_		*r*_ac_	
		CV1	CV2	CV1	CV2
DS-CC	DS-joint	0.457	0.443	0.528	0.511
FAI-BLUP	DS-joint	0.500	0.465	0.578	0.537
DS-2018	DS -2019	0.592	0.536	0.688	0.623
DS -2018	DS -2020	0.343	0.297	0.383	0.332
DS -2019	DS -2018	0.400	0.261	0.504	0.329
DS -2019	DS -2020	0.455	0.300	0.509	0.335
DS -2020	DS -2018	0.371	0.264	0.467	0.333
DS -2020	DS -2019	0.670	0.541	0.779	0.623

Using single-field DS data to predict the response of a novel accession in a different field season revealed varying results. Training in 2018 and validating in 2019 data showed high predictive abilities and accuracies as shown in **Table 4**, indicating that the model could accurately capture the year-to-year environmental variance. The models trained in 2020 and validated in 2019 exhibited the highest predictive ability and accuracy. However, training models on DS-2019 and validating on DS-2018 decreased the predictive ability and accuracy. In summary, while the incorporation of the M×E matrix into the GBLUP model did not significantly influence the average predictive abilities and accuracies under CV1 scenario, it improved the predictive abilities under CV2 scenario by 11% on average (**Supplementary Figure 2**). This underscores the potential for the GBLUP model, with the M×E interaction, to predict disease resistance with considerable accuracy, especially when data can be validated with DS collected in field seasons with high and homogeneous rust infestation levels.

## 4 Discussion

The integration of quantitative genomic methodologies into plant breeding has opened a new era for variety development, characterized by reduced cycle times and cost savings through diminished reliance on extensive phenotyping (Crossa et al., 2017). GS has gained attraction in legume breeding but its accurate prediction of complex traits, such as disease resistance with quantitative inheritance and strong environmental interactions, remains a challenge and a significant barrier for its effective integration into routine plant breeding workflows (Rubiales et al., 2021). Our assessment of GS potential for pea rust tolerance relied on a genotype sample size comparable with that of other training sets for pea GS (Annicchiarico et al., 2019; Annicchiarico et al, 2017b; Tayeh et al., 2015), a thorough multi-environment phenotyping, and 26,045 high-quality polymorphic Silico-DArT markers covering all pea chromosomes after thorough data filtering (**Figure 1**). The average polymorphism information content (PIC) value of these markers was 0.29 (Rispail et al., 2023), indicating a moderate level of informativeness according to Hildebrand et al. (1992) classification although it was higher than recent studies on genetic diversity in pea using the same DArT approach, where the average PIC was 0.26 (Brhane and Hammenhag, 2024). In addition, the fairly weak genetic structure reported for our pea germplasm collection by Rispail et al. (2023) was beneficial, as it may increase the accuracy in cross-validation schemes of GS model validation and application.

GEI is a significant factor influencing rust DS in pea, as shown in field studies by Das et al. (2019) and Osuna-Caballero et al. (2022). Despite the complexity introduced by GEI, the phenotypic and genotypic correlations of DS under field conditions and the stability of selected accessions across environments allowed to consolidate three individual field evaluations into one value (DS-joint). The DS-joint reduces the effect of GEI and allows researchers to extract valuable information about accessions more effectively, making it a suitable solution for traits highly affected by GEI such as rust DS (Gonzalez-Barrios et al., 2019). The BLUP values derived from the DS-joint successfully integrated the variance of the three environments while retaining high correlation with each environment. This DS-joint dataset, which reflects analogous climatic and edaphic conditions, is expected to act as a robust validation set for models trained on controlled conditions, as confirmed by other studies (Lado et al., 2016). Nevertheless, the shrinkage effect associated with random effects can be over- or underestimated in the mixed models using MET data (Olivoto et al., 2019). Therefore, GEI were explored in more details through AMMI to evaluate the most promising material derived from Osuna-Caballero et al. (2022) studies. AMMI analysis using MET data targeted two main goals: (i) to understand complex GEI, including selected genotypes to exploit narrow adaptations, and (ii) to gain in accuracy to improve recommendations, repeatability, selections, and genetic gains (Gauch, 2013).

Field phenotyping of extensive collections is critical for elucidating GEI, a cornerstone for the successful deployment of GS schemes (Budhlakoti et al., 2022). Nonetheless, this necessity stands in contrast to the associated high cost and intensive labor. In this study, we have explored the potential of using controlled condition data to predict rust severity observed under field conditions, where disease-related parameters can be measured with greater accuracy. While some multi-trait GS models that incorporate various parameters for model training and validation have reported promising results (Gill et al., 2021), others have found that the improvements are not consistently marked across different traits (Jarquín et al., 2014). Our study introduced an alternative approach that consolidates traits measured under controlled conditions into a single index: FAI-BLUP (Rocha et al., 2018). This index, similar to MGDI, accounts for the multicollinearity among parameters, yielding more favorable outcomes than traditional indices founded on linear models (Rocha et al., 2018; Gonzalez-Barrios et al., 2019). Accordingly, the FAI-BLUP index showed the best predictive abilities of the controlled condition traits in the intra-environment GS scheme regardless of the model, highlighting its efficacy, and validating its application for other GS scenarios. Other authors who evaluated the consistency of multi-trait GS models obtained lower predictive abilities compared to the application of the FAI-BLUP index proposed here in intra-environment and across-environment configurations (Ward et al., 2019; Gill et al., 2021).

In the initial GS scheme, the predictive abilities of three models were evaluated for each trait within controlled and field conditions. While rrBLUP and BL models showed comparable predictive abilities under controlled conditions, GBLUP excelled under field conditions, boasting superior predictive abilities as previously shown in other related studies (Wang et al., 2018; Nazzicari and Biscarini, 2022). Notably, this enhancement in predictive performance was not mirrored for the FAI-BLUP index, which exhibited uniform prediction abilities across all three models. Additionally, the predictive ability of GBLUP was diminished for the DS-joint assessment in comparison when DS-2019 data set was used to validate the across-environment models but the results improved in all the other configurations. These discrepancies may be attributed to the distinct methodologies and assumptions inherent to each model. GBLUP, like rrBLUP, assumes that traits are influenced by many genes with small effects. However, the GBLUP advantage emerges when considering the population structure and genetic relationships, which are critical factors under field conditions (Habier et al., 2007). Field conditions may reflect more accurately the rust DS across the complete lifecycle of the plant, whereas controlled conditions can only evaluate the initial rust disease cycle on seedlings. Such controlled settings may not fully capture the phenotypic expressions that characterize each sub-population—such as leaf size and overall plant size—that could impact disease variation. Conversely, in the field, these sub-population traits are considered by the GBLUP model, leading to more accurate predictions. This observation aligns with other studies where GBLUP has been preferred over rrBLUP and BL, particularly when modelling traits under field conditions that are influenced by population structure (Guo et al., 2014; Roorkiwal et al., 2016).

In the following GS framework, which is more relevant for plant breeding, models were cross trained using data from two distinct conditions, and predictions were made for the validation set, which was also part of the training set (CV1). This strategy is particularly informative when the environments are homogeneous and exhibit strong interrelatedness. Indeed, our results confirmed that environments with greater genetic or phenotypic similarity can yield higher predictive abilities, as reported in earlier pea studies by Carpenter et al. (2018) and Crosta et al. (2023). Consistently, the GBLUP model provided the most robust predictions, achieving a predictive accuracy of 0.730 when trained on DS-2020 and validated on DS-2019, and 0.577 when employing the FAI-BLUP index for training and validation on DS-joint. This finding, which highlighted the enhanced predictive potential of a multi-trait index over single traits measured under controlled conditions, can represent a methodological progress over other GS research focused on pea diseases employing a similar cross-validation scheme (CV1) (Carpenter et al., 2018). With this method, we achieved higher prediction accuracies than those reported for rust resistance in wheat, which ranged from 0.33 to 0.44 using the same validation strategy and the GBLUP model (Daetwyler et al., 2014).

In the context of plant breeding, the most valuable and challenging scheme is the one that allows the prediction of phenotypic values of novel lines in untrained environments, a configuration referred to as CV2. In this study, CV2 assessment for rust resistance in pea mirrored the pattern seen in CV1, albeit with lower r_ab_ and r_ac_ values. This outcome aligns with previous studies that documented a decline in predictive performance from CV1 to CV2 across legumes (Carpenter et al., 2018; Gill et al., 2021). Despite this drop, the GBLUP model continued to exhibit moderate and still useful predictive abilities and accuracies, outperforming other models. The most effective approach to predict rust response in the field with controlled conditions data was to train models with the FAI-BLUP index and validate them on DS-joint. By contrast, to predict rust responses under field conditions with DS collected under field condition, the most accurate predictions were obtained when DS-2019 was used as validation dataset. This strategy achieved higher predictive values for plant disease resistance compared to other GS approaches using similar strategies (Rutkoski et al., 2011; Juliana et al., 2017), confirming the utility of our models in practical breeding applications for rust resistance.

In the final GS arrangement, we revisited the CV1 and CV2 cross-validation scenario of GBLUP model, which consistently provided superior predictive abilities and accuracies for the evaluated traits. In this iteration, we introduced the DArT-seq marker matrix’s interaction with the environments (M×E) as covariate model (Lopez-Cruz et al., 2015). This addition confirmed the critical role of GEI effects, enabling refined adjustments to enhance the model predictive abilities, as demonstrated in other studies targeting disease resistance in pea (Carpenter et al., 2018). This modification is especially beneficial for traits with complex genetic architectures such as rust resistance in pea, which are shaped by the interplay of genetic and environmental factors. By incorporating the M×E interaction, the model gains the capacity to account for the unique expression of genetic markers across different environments, a factor essential for the accurate prediction of phenotypes in variable conditions (Cuevas et al., 2016). As other researchers have shown for rust disease in other species, modifying the GBLUP model to include the M×E matrix empowers it to discern marker effects that may be prominent in one environment but not others, an aspect that is critically important in the CV2 scheme, where validation occurs in an environment not represented in the training dataset (Lopez-Cruz et al., 2015; Fois et al., 2021). Accounting for these interactions has generated more precise predictions in new environments, improving accuracy by up to 11% in this study. We suggest that integrating the M×E effect might reduce model bias. Without this interaction, the model might be overfitting the conditions of the training set, particularly in the CV1 scheme. Thus, the inclusion of the M×E covariate is instrumental to generalize the model, ensuring stability, and enhancing the accuracy of predictions across a spectrum of environmental scenarios.

The practical implications of our findings can be significant in pea breeding for a trait, such as rust resistance, which is genetically complex and affected by GEI. The adoption of GS based on GBLUP with M×E interactions can decrease the need for extensive phenotyping, which is often resource-intensive and environmentally constrained. The use of the FAI-BLUP index further refined our predictions, by integrating multiple traits associated with disease resistance under controlled conditions, thereby providing a more holistic view of the genetic potential of each accession. This multi-trait integration strategy may prove useful for other complex traits in plant breeding. The optimized model is already being used to select lines for rust resistance for future field trials in our pea breeding program, which will serve to continue enriching the model as well as to continue increasing the predictive abilities. In conclusion, our study not only reaffirms the efficacy of GS in plant breeding but also advances our understanding of how to effectively model complex traits. The insights gained here have broader applications in the field of agricultural genetics, providing a roadmap for harnessing genomic tools to accelerate the development of crop varieties that are resilient to diseases and adaptable to varying environmental conditions. The integration of advanced genomic tools, such as those explored in this study, will be instrumental in meeting the growing challenges of global food security and sustainable agriculture.

## Data availability statement

Publicly available datasets were analyzed in this study. This data can be found here: The DArTseq marker datasets analyzed in this study can be found in the Zenodo repository, https://zenodo.org/records/7180467. The phenotypic datasets are available in the GitHub repository, https://github.com/SalvaOsuna/Rust-collection.git.

## Author contributions

SO-C: Formal analysis, Investigation, Methodology, Writing – original draft. DR: Conceptualization, Funding acquisition, Supervision, Writing – review & editing. PA: Writing – review & editing. NN: Methodology, Writing – review & editing. NR: Conceptualization, Supervision, Writing – review & editing.
