# Supplementary material for: Genomic prediction for rust resistance in pea
Source: Front Plant Sci. 2024 Jul 23;15:1429802. doi: 10.3389/fpls.2024.1429802 (PMC11300365; doi:10.3389/fpls.2024.1429802)
Supplement: Supplementary file 1 [file Image_1.pdf]

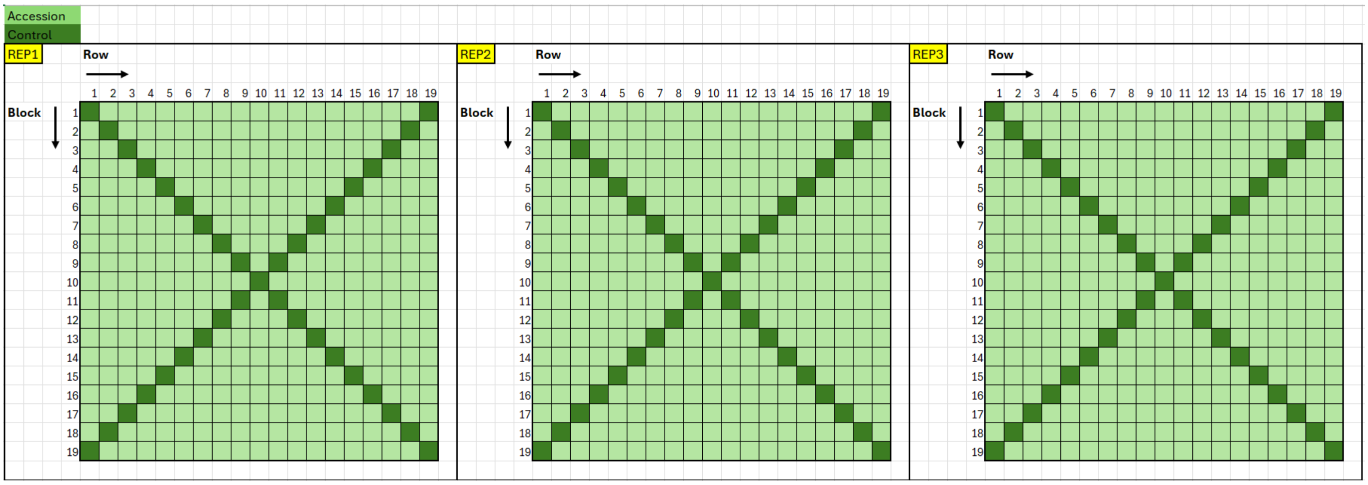

Supplementary Figure 1. Graphical representation of the alpha lattice experimental design. Each replicate contains 19 blocks, and each block contains 17 accessions out of the total 324 (except for block nº 10), making them incomplete blocks. Same accession appears once per block, but the overall design ensures that each accession appears in different blocks across replicates. Within each block, the assignment of treatments would be randomized.
