# Supplementary material for: Genomic prediction for rust resistance in pea
Source: Front Plant Sci. 2024 Jul 23;15:1429802. doi: 10.3389/fpls.2024.1429802 (PMC11300365; doi:10.3389/fpls.2024.1429802)
Supplement: Supplementary file 2 [file Image_2.pdf]

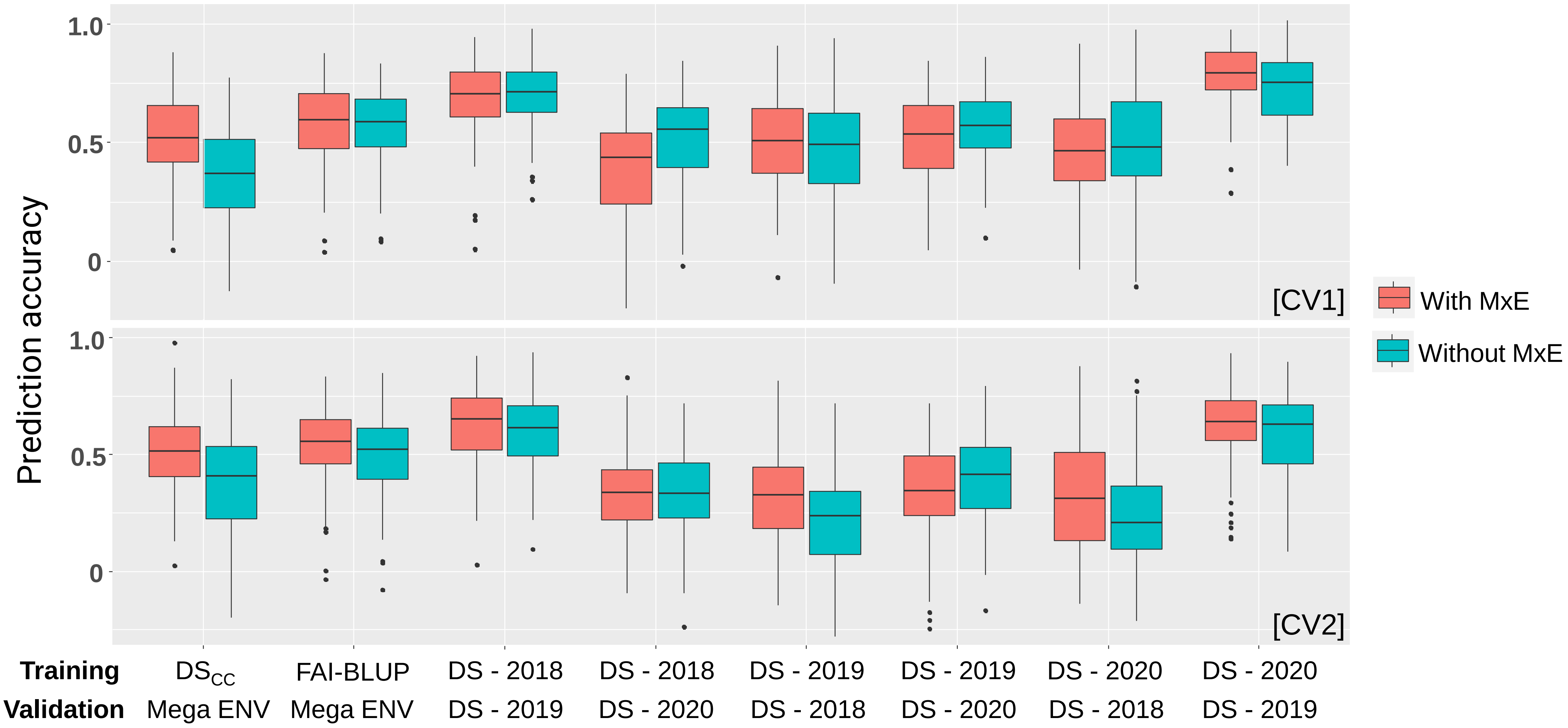

Supplementary Figure 2. Prediction accuracy of GBLUP model in two cross-validation schemes (CV1 at the top and CV2 at the bottom) based in the training/validation test described at the bottom of each boxplot.
