# Supplementary material for: Genomic prediction for rust resistance in pea
Source: Front Plant Sci. 2024 Jul 23;15:1429802. doi: 10.3389/fpls.2024.1429802 (PMC11300365; doi:10.3389/fpls.2024.1429802)
Supplement: Supplementary file 3 [file Table_1.pdf]

Source	Df	Sum Sq	Mean Sq	F value	Pr(>F)	Proportion	Accumulated
**ENV**	2	68194	34097	147.43	6.19E-60	NA	NA
**REP(ENV)**	6	139557	23260	100.57	5.08E-109	NA	NA
**BLOCK(REP*ENV)**	162	81240	501	2.17	4.49E-14	NA	NA
**GEN**	323	277957	861	3.72	1.39E-69	NA	NA
**GEN:ENV**	646	485286	751	3.25	5.04E-84	NA	NA
**PC1**	324	385781	1191	5.15	0.00E+00	79.3	79.3
**PC2**	322	100731	313	1.35	1.00E-04	20.7	100
**Residuals**	1764	407964	231	NA	NA	NA	NA
**Total**	3549	1946710	549	NA	NA	NA	NA

Supplementary Table 1. AMMI analysis table. Two interactions principal component axis (IPCA) were fitted and significant at 5% probability error.
